# Human seminal plasma stimulates the migration of CD11c+ mononuclear phagocytes to the apical side of the colonic epithelium without altering the junctional complexes in an *ex vivo* human intestinal model

**DOI:** 10.3389/fimmu.2023.1133886

**Published:** 2023-03-22

**Authors:** Marco Baratella, Valeria Iannone, Mariangela Cavarelli, Chiara Foglieni, Paola Viganò, Christiane Moog, Ugo Elmore, Silvia Nozza, Massimo Alfano, Andrea Salonia, Stefania Dispinseri, Gabriella Scarlatti

**Affiliations:** ^1^ Viral Evolution and Transmission Group, Division of Immunology, Transplantation, and Infectious Diseases, Istituti di Ricovero e Cura a Carattere Scientifico (IRCCS) Ospedale San Raffaele, Milan, Italy; ^2^ Center for Immunology of Viral, Auto-immune, Hematological and Bacterial diseases, Commissariat à l'énergie atomique et aux énergies alternatives (CEA), Université Paris-Saclay, Inserm, Paris, France; ^3^ Cardiovascular Research Center, Istituti di Ricovero e Cura a Carattere Scientifico (IRCCS) Ospedale San Raffaele, Milan, Italy; ^4^ Reproductive Sciences Laboratory, Gynecology/Obstetrics Unit, Istituti di Ricovero e Cura a Carattere Scientifico (IRCCS) Ospedale San Raffaele, Milan, Italy; ^5^ INSERM U1109, Fédération de Médecine Translationnelle de Strasbourg, Université de Strasbourg, Strasbourg, France; ^6^ Department of Gastrointestinal Surgery, Istituti di Ricovero e Cura a Carattere Scientifico (IRCCS) Ospedale San Raffaele, Milan, Italy; ^7^ University Vita-Salute San Raffaele, Milan, Italy; ^8^ Division of Infectious Diseases, Istituti di Ricovero e Cura a Carattere Scientifico (IRCCS) Ospedale San Raffaele, Milan, Italy; ^9^ Division of Experimental Oncology, Unit of Urology, Istituti di Ricovero e Cura a Carattere Scientifico (IRCCS) Ospedale San Raffaele, Milan, Italy

**Keywords:** HIV-1 transmission, intestinal mucosa, human seminal plasma, mononuclear phagocytes, CD11c+ cells migration

## Abstract

**Introduction:**

Human immunodeficiency virus type 1 (HIV) transmission mostly occurs through the genital and intestinal mucosae. Although HIV-1 transmission has been extensively investigated, gaps remain in understanding the initial steps of HIV entry through the colonic mucosa. We previously showed that HIV can selectively trigger mononuclear phagocytes (MNP) to migrate within colonic epithelial cells to sample virions. Mucosal exposure to human seminal plasma (HSP), rich in pro- and anti-inflammatory cytokines, chemokines and growth factors, may as well induce alterations of the colonic mucosa and recruit immune cells, hence, affecting pathogen sampling and transmission.

**Methods:**

Here, we studied the role of HSP on the paracellular intestinal permeability by analyzing the distribution of two proteins known to play a key role in controlling the intestinal barrier integrity, namely the tight junctions-associated junctional adhesion molecule (JAM-A) and the adherents junction associated protein E-cadherin (E-CAD), by immunofluorescence and confocal microscopy. Also, we evaluated if HSP promotes the recruitment of MNP cells, specifically, the CD11c and CD64 positive MNPs, to the apical side of the human colonic mucosa. At this scope, HSP of HIV-infected and uninfected individuals with known fertility status was tested for cytokines, chemokines and growth factors concentration and used in an *ex vivo* polarized colonic tissue culture system to mimic as closely as possible the physiological process.

**Results:**

HSP showed statistically significant differences in cytokines and chemokines concentrations between the three groups of donors, i.e. HIV infected, or uninfected fertile or randomly identified. Nevertheless, we showed that in the *ex vivo* tissue culture HSP in general, neither affected the morphological structure of the colonic mucosa nor modulated the paracellular intestinal permeability. Interestingly, CD11c+ MNP cells migrated to the apical surface of the colonic epithelium regardless, if incubated with HIV-infected or -uninfected HSPs, while CD64+ MNP cells, did not change their distribution within the colonic mucosa.

**Discussion:**

In conclusion, even if HSP did not perturb the integrity of the human colonic mucosa, it affected the migration of a specific subset of MNPs that express CD11c towards the apical side of the colonic mucosa, which in turn may be involved in pathogen sampling.

## Introduction

Human immunodeficiency virus 1 (HIV) infects millions of people worldwide each year (https://www.unaids.org/sites/default/files/media_asset/UNAIDS_FactSheet_en.pdf). Mucosal tissues are the primary site of natural HIV transmission and a major reservoir for viral replication (1). In fact, HIV is transmitted through sexual intercourse by crossing epithelial barriers at mucosal surfaces of the genital and anorectal tracts. HIV can cross both genital and colonic mucosa although epithelial cells of these tissues do not express HIV receptors (2). The first events of HIV infection in the human intestinal tract have been extensively investigated (3, 4), but gaps remain in understanding the initial steps of the HIV entry through the colonic mucosa. HIV from male ejaculate must bypass innate and adaptive immune factors, travel through the genital epithelium, and establish infection in underlying CD4+ T cells.

In the intestine, a single layer of polarized intestinal epithelial columnar cells (IECs) tightly connected by junctional complexes (5, 6), represent the first physical line of defense from invading pathogens of the intestinal mucosa (7). The paracellular permeability is mainly regulated by tight junctions (TJ) and adherent junctions (AJs) (8). Two main proteins, the TJs-associated junctional adhesion molecule A (JAM-A) and E-cadherin (E-CAD), are crucial in maintaining the homeostasis and in promoting the assembly of tight and adherent junctions respectively (9, 10). To bypass the epithelial barrier, HIV or other pathogens may take advantage of the disruption of such complexes, for example induced by the human seminal plasma (HSP). HSP is thought to have the potential to dysregulate the junctions on IECs (11) *via* signaling molecules that may interact with those proteins that regulate the paracellular permeability (12). In addition, while HSP is known to modify the immune cell distribution of the vaginal tract *in vivo* and, within hours, to induce recruitment of macrophages (MCs), dendritic cells (DCs) and granulocytes into the endometrial stroma and lumen (13), less is known about its effect in the human colonic mucosa. We previously described the migration of innate immune cells through the human colonic epithelium in response to HIV in an *in vitro* cell- and *ex vivo* colonic tissue-culture system and suggested that these immune cells may be exploited by the virus as carriers to cross the epithelium and reach in the mucosa those target cells relevant for virus replication (14). Whether HSP of HIV positive or negative individuals may differently modulate the migration of mononuclear phagocytes (MNP) toward the apical side of the colonic mucosa is to be defined. Whether the fertility status may play a role as well is also unclear. Indeed, studies have shown that cytokines and chemokines may differ significantly between fertile and infertile men, as well as between HIV-positive and HIV-negative men. For example, higher levels of pro-inflammatory cytokines such as TNF-α and IL-1β have been found in the semen of infertile men, while lower levels of chemokines such as CXCL12 have been associated with infertility (15, 16). HIV-positive men have also been shown to have altered cytokine and chemokine profiles in their seminal plasma (17). These findings suggest that the cytokine and chemokine profiles in seminal plasma may vary greatly between these three groups.

We have previously shown that CD11c+ but not CD68+ MNP migrate in response to HIV, suggesting that DCs more than MCs would be involved in transepithelial migration and viral transport (14, 18). However, a thorough characterization of innate cells of the lower intestinal mucosal tract in human and non-human primates (NHP) (19, 20), showed that MCs can be distinguished from DCs by the specific expression of the high-affinity IgG receptor FcgRI, CD64 (21), and usually by the lack of expression of CD11c surface marker unless when in an immature state (19). Furthermore, colonic MNP express chemokine receptors on their surface (19, 22), and thus, a chemotactic gradient of several components contained in HSP may influence their migration. Indeed, in an *in vitro* Caco-2 cell monolayer/DC co-culture model, we showed that the chemokine CCL5, induced DCs migration through engagement of their receptors (14). In addition, in an *ex vivo* NHP colonic model, seminal plasma from leukocytospermic macaques induced the intraepithelial recruitment of HLA-DR+ CD11c+ cells and facilitated SIV transmission (23). If cell migration may be affected by differences in cytokine, chemokines and growth factor concentrations in HSP of HIV infected and uninfected individuals is still to be defined.

In the current study, we aimed to elucidate the effect of HSP in altering the gut barrier functions and on the migration of innate immune cells in the human colon. We found differences in concentration of cytokine, chemokines and growth factor concentrations in HSP from HIV infected and uninfected individuals with known fertility status. However, none of the HSP was able to perturb IECs paracellular permeability. CD11c+ MNP cells infiltrated in the epithelial layer of the mucosa, mostly within the epithelium of the crypts irrespectively whether stimulated by HIV infected or uninfected HSPs, while CD64+ MNP did not show any clear pattern of migration within the colonic tissue.

## Materials and methods

### Study subjects and ethics permissions

Forty-seven ejaculates were obtained from 14 HIV-positive, 12 known fertile, and 21 randomly identified subjects at the IRCCS Ospedale San Raffaele, Milan, Italy. HIV infected donors (**Table 1**), followed at the Division of Infectious Diseases, at the time of ejaculate collection, had a median age of 37.5 years (min 28; max 55) and were diagnosed since a median time of 5.5 years (min 2; max 20). The median plasma HIV load was 2,468 copies/mL (min 45; max 119,116) and the median CD4+ T cell count was 636.5/mm^3^ (min 371; max 10,027). All except one were naïve for antiretroviral therapy. Fertile HIV negative subjects had fathered at least one child, spontaneously conceived, with a time-to-pregnancy within 12 months. Donors (age 30 to 42 years, median 36) were recruited *via* their partners who had been expectant and new mothers at Department of Obstetrics and Gynecology, IRCCS San Raffaele Hospital. Semen analyses, including count of leukocytes in the seminal plasma (min 0.03; max 0.25 million/mL; median 0.1 million/mL), were based on 2010 WHO reference criteria (24). Additional twenty-one ejaculates were anonymously chosen from individuals attending the Centro Scienze della Natalità. No data were available from these donors though most probably HIV negative and with limited fertility as programmed for *in vitro* fertilization procedure. An informed consent agreeing to supply their own anonymous information and biological specimens was obtained from all individual participants included in the study. Collection of ejaculates obtained approval by the IRCCS Ospedale San Raffaele Ethics Review Board for HIV infected subjects (HIVSPERM, protocol number 5/INT/2014, 2014/02/06), for subjects followed at Centro Scienze della Natalità (BC-GINEOS protocol) and for the fertile subjects (URIMALES-2016, further amended on March 2018). For the biobanking of material of fertile subjects the authorization protocol URI001-2010, further amended on December 2015 was obtained.

**Table 1:** Clinical parameters of HIV positive donors.

Donor code	Age (Years)	CD4/mm^3^	CD8/mm^3^	CD4/CD8 ratio	HIV-1 plasma load (copies/Ml)
1	36	421	1360	0.31	2155
2	25	10027	989	1.04	1026
3	31	511	1311	0.39	2780
4	52	778	1731	0.45	18407
5	33	371	2201	0.17	84545
6	31	1114	1002	1.11	1957
7	29	624	1342	0.36	1818
8	46	416	1054	0.39	2063
9	40	563	1115	0.59	30731
10	37	807	1474	0.55	119166
11	51	818	845	0.97	45
12	45	649	1102	0.59	48526
13	28	604	465	0.8	3680
14	26	938	420	2.23	45

Clinical parameters including age, CD4+ and CD8+ T cells (cells/mm^3^), CD4/CD8 ratio and HIV plasma load (copies/mL) of HIV-1 positive donors.

### Sampling and processing of HSP

Semen was collected at the hospital and processed within 1 hour as follow. Ejaculates were centrifugated at 3000 rpm for 10 minutes in order to separate the seminal plasma. HSP of each group of donors was pooled by mixing 100 μL from each single donor and named HIV-positive pool when from HIV-infected donors, Fertile pool when from the fertile donors, and Random pool when obtained from the randomly identified donors of the Infertility clinic. The HSP from the 12 fertile donors was also kept separate. HSP was stored in aliquots at minus 80°C until use.

### HIV load determination in plasma and HSP

HIV RNA concentration was quantified in plasma and HSP pool of HIV infected donors using COBAS TaqMan HIV-1 test V2.0 according to manufacturer’s instructions (Roche Diagnostics, Monza, IT). The assay has a lower limit of detection of 20 HIV RNA copies/mL and a linear range of detection up to 20 – 1 x 10^7^ copies/mL.

### Measurement of cytokines and chemokines in HSP

The concentration of cytokines and chemokines was measured in duplicates of the HSP by Human Magnetic Luminex Screening Assay (Labospace Srl, Milano, IT). Samples were processed following the manufacture instruction by diluting the HSP at 1:2 in Phosphate Buffer Saline (PBS; Lonza, Mi, IT) for IL-4, IL-10, IL-13, IL-1b, IL-1ra, IL-2, IL-6, IL-8, IL-15, IL-17A, TNFα, IFNγ, CCL2, CCL3, CCL4, IP-10, CXCL12, CTACK, CXCL9 and GM-CSF; at 1:50 for CCL5 and at 1:15 for TGF-β1, TGF-β2 and TGF-β3 determination.

### Human colonic *ex vivo* culture model

The Department of Gastrointestinal Surgery of the IRCCS Ospedale San Raffaele, provided colon fragments from patients undergoing surgery for non-invasive colon cancer, under the MUCHIV protocol approved by IRCCS Ospedale San Raffaele Ethics Review Board (protocol number #35 06/02/2014 and extension #40 09/11/2015). Specimens, collected far from the pathological area, of the rectosigmoid segment or descending colon were approximately 2 cm^2^ surgical sections and placed in 0.9% saline solution to be processed within 30 minutes from the excision, as described previously (14). Briefly, after abundant washes with PBS tissue specimens were transferred into complete RPMI medium (Euroclone, Milan, IT) supplemented with 10% FCS, 100 U/mL penicillin/streptomycin, 1% glutamine, 1% NEAA, 1% Na-pyruvate and 1% HEPES buffer 1 M. Tissue cylinders of 8.0 mm diameter were cut with a biopsy punch (Stiefel, Laboratories, Inc. North Carolina, USA), and placed on a sponge support (BioOptica, Milan, IT) with the submucosa facing the sponge. A polystyrene cylinder of 8.0 mm diameter (Sigma–Aldrich, St. Louis, MO, USA) was sealed with veterinary glue (3M Vetbond, St. Paul, MN, USA) onto the borders of the apical surface of the mucosa. Specimens were placed in a 60 mm center-well organ culture dish (BD Falcon, San Diego, CA, USA) containing 1 mL of complete medium.

Mucosal explant cultures were treated apically for 30 and 90 minutes with either HSP diluted at 1/10 in 10% FCS RPMI medium or only 10% FCS RPMI medium (control medium). After incubation, the apical supernatant was removed, and specimens were rinsed three times with 100 µL of PBS.

### Immunohistochemistry, immunofluorescence and confocal microscopy

Tissues were fixed overnight at 4°C in PBS containing 4% paraformaldehyde (PFA) (Sigma-Aldrich, Taufkirchen, DE), and then cryoprotected for 6 hours at 4°C in 10% sucrose (Sigma-Aldrich, Taufkirchen, DE). Thereafter the cylinder was carefully removed with forceps, the tissue embedded in killik (BioOptica, Milan, IT) and snap-frozen in isopentane and liquid nitrogen. Ten μm-thick sections, 100 μm far from each other were cut with a Leica CM1850 cryostat (Leica Microsystems GmbH, Wetzlar, DE).

Morphological tissue preservation was verified by brightfield microscopy (Olympus BX40) after Hematoxylin-Eosin staining on three randomly chosen section. For immunofluorescence and confocal microscopy, the sections were placed on microscope glass slides (Superfrost plus, Thermo scientific, Waltham, MA, USA), rehydrated in PBS for 1 hour, permeabilized with Triton X-100 0.5% in PBS for 45 minutes, incubated with 3% BSA in PBS for 30 minutes and stained with primary antibodies diluted in PBS with 0.5% BSA overnight at 4° C. Slides were then washed with PBS three times for 10 minutes and incubated with a fluorochrome conjugated appropriate secondary antibody for 45 minutes at room temperature in the dark.

The primary antibody used were: mouse anti human JAM-A FITC conjugated (clone BV16, Hycult biotech, Uden, NL), mouse anti human E-Cadherin (clone 36/E, BD Bioscience, Franklin Lake, NJ, USA), rabbit anti human CD11c (ab52632, ABCAM, Cambridge, UK), and mouse anti human CD64 (ab140779, ABCAM, Cambridge, UK). The secondary antibodies were: goat anti-mouse IgG Alexa Fluor 488 conjugated or goat anti-rabbit IgG Alexa Fluor 546 conjugated (Thermo Fisher,Waltham MA, USA). Nuclei were counterstained with DAPI at the concentration of 0.416 µg/mL (Thermo Scientific, Waltham, MA USA) for 10 minutes at room temperature.

Coverslips were mounted on slides with FluorSave reagent (Calbiochem, Vimodrone, MI, IT) and examined by a confocal laser scanning microscope (Leica TCS SP5) or a fluorescent microscopy (Olympus BX40). Representative images from three independent experiments were acquired. For each processed colonic tissue at least ten images were analyzed by Fiji (Image J) software and GIMP 2.10.30.

### Statistical analysis

All data visualization and statistical analyses were carried out using Prism v9.2.0 software (GraphPad software, La Jolla, USA). The statistical significance was tested using unpaired t-test. P values ≤0.05 were considered significant, ∗p < 0.05, ∗∗p < 0.01, ∗∗∗p < 0.001.

## Results

### Concentration of cytokines, chemokines and granulocyte-macrophage colony-stimulating factor in HIV positive HSP is different from HIV negative HSP pool

We evaluated the concentration of anti-inflammatory cytokines (IL-4, IL-10, TGF-ß1, TGF-ß2, TGF-β3 and IL-13), pro-inflammatory cytokines (IL-1β, IL-1ra, IL-2, IL-6, IL-8, IL-15, IL-17A, TNFα and IFNγ), chemokines (CCL2, CCL3, CCL4, IP-10, CXCL12, CCL5, CTACK and CXCL9) and granulocyte-macrophage colony-stimulating factor (GM-CSF) in HSP samples of three pools from 12 HIV negative fertile, from 21 HIV negative random or from 14 HIV positive donors. HSP of each group of donors was pooled by mixing 100 μL from each individual donor and subsequentially assessed for analytes.

**Figure 1:**
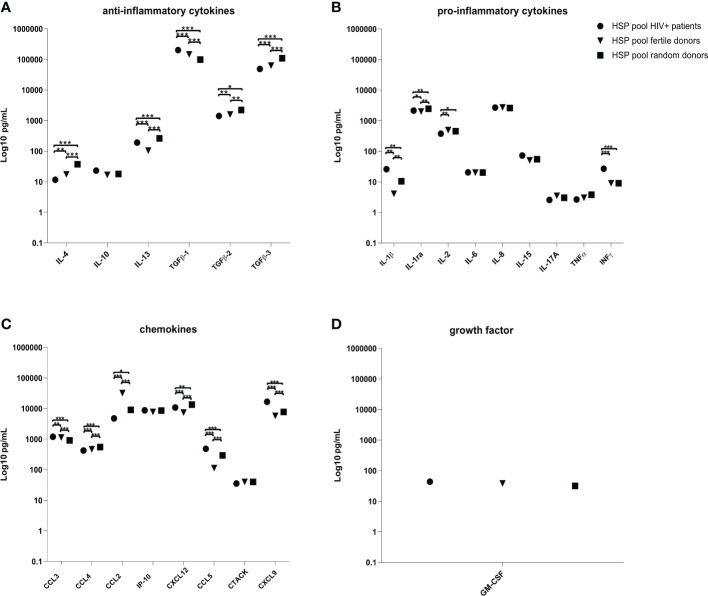
Cytokines, chemokines and GM-CSF quantification in HSP pools. Concentrations (pg/mL) of ant-inflammatory cytokines **(A)**, pro-inflammatory cytokines **(B)**, chemokines **(C)** and GM-CSF **(D)** in the three pools of HSP samples obtained from 12 HIV negative fertile, from 21 HIV negative random or from 14 HIV positive donors. HSP of each group of donors was pooled by mixing 100 μL from each individual donor and subsequentially assessed for analytes. LOD, limit of detection. ∗p < 0.05, ∗∗p < 0.01, ∗∗∗p < 0.001.

While the concentration of GM-CSF was similar in all HSP pools, several cytokines and chemokines showed significant differences between the three pools (**Figure 1** and **Supplementary Table**). HSP pooled from HIV positive individuals (a) compared to HSP pools from fertile (b) and random (c) donors, showed statistically significant higher concentrations of TGF- ß1 ((b) p = 0.0001, (c) p = 0.0001), IL-1ß ((b) p = 0.0021, (c) p = 0.0061), IFN-γ ((b) p = 0.0004, (c) p = 0.0004), CCL3 ((b) p = 0.0035, (c) p = 0.0001), CCL5 ((b) p = 0.0002, (c) p = 0.0009) and CXCL9 ((b) p = 0.0001, (c) p = 0.0001). While it exhibited a significantly lower concentration of IL-4 ((b) p = 0.0071, (c) p = 0.0001), IL-1ra ((b) p = 0.0161, (c) p = 0.0022), TGF-ß2 ((b) p = 0.0246, (c) p = 0.0016), TGF-β3 ((b) p = 0.0003, (c) p = 0.0001), CCL4 ((b) p = 0.0007, (c) p = 0.0001) and CCL2 ((b) p = 0.0008, (c) p = 0.0162). IL-2 showed a significant lower concentration in HSP pooled from HIV positive individuals compared to HSP pools from fertile (p = 0.0032) and random (p = 0.0022) donors.

IL-13 (p = 0.0002) and CXCL12 (p = 0.0008) were significantly higher in the HIV positive donor pool compared to the fertile donor pool, but lower compared to the random pool (p = 0.0002, p = 0.0013). HSP pooled from HIV negative fertile donors compared to HSP pools from random donors showed statistically significant higher concentrations of TGF-ß1 (p = 0.0001), IL-2 (p = 0.0297), CCL3 (p = 0.0001), CCL2 (p = 0.001) and statistically significant lower concentration of IL-4 (p = 0.0008), IL-13 (p = 0.0001), TGF-ß2 (p = 0.0039), TGF-β3 (p = 0.0001), IL-1 ß (p = 0.0061), IL-1ra (p = 0.0013), CCL4 (p = 0.0002), IP-10 (p = 0.033), CXCL12 (p = 0.0001), CCL5 (p = 0.0002) and CXCL9 (p = 0.0001).

### Quantification of cytokines, chemokines and GM-CSF of twelve HSP samples from fertile donors

**Figure 2:**
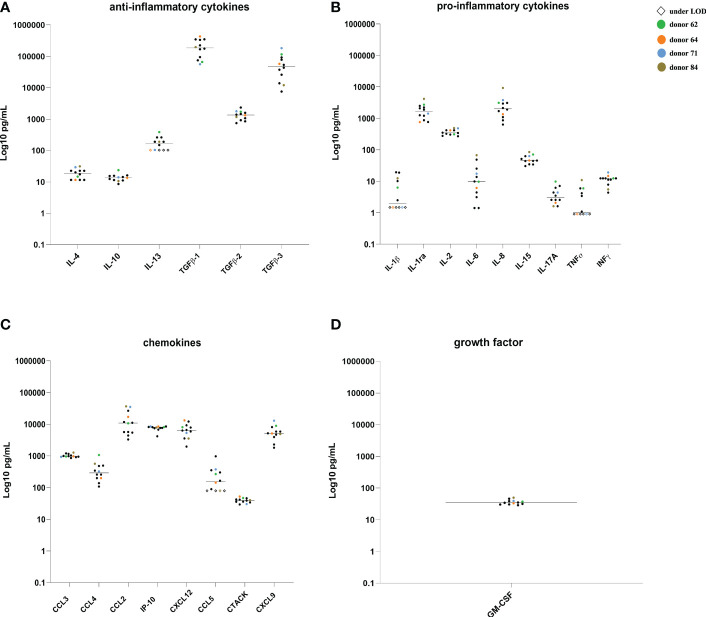
Cytokines, chemokines and GM-CSF quantification in the HSP from 12 fertile donors. Concentrations (pg/mL) of ant-inflammatory cytokines **(A)**, pro-inflammatory cytokines **(B)**, chemokines **(C)** and GM-CSF **(D)** in single samples from HIV negative fertile subjects. Colored symbols identify HSP of four donors subsequently used for colonic *ex vivo* cultures. LOD, limit of detection.

Cytokines, chemokines and GM-CSF were measured singularly in twelve HSP from fertile donors (**Figure 2**). Most of the determinations were within 3- to 10-fold difference within each substance, while a vast range (100-fold) was identified for TGFβ-3 and IL-6. Only IL-3, IL-1β, TNF-α and CCL5 included a group of HSPs under the detection limit of the assay. However, the HSP’s concentration were in the range of published values (25, 26).

Three donors showed a higher concentration of some analytes compared to the average concentration of the samples, namely TGF-β3 (116,506.36 pg/mL), CCL4 (1,064.33 pg/mL) and CXCL9 (8,909.65 pg/mL) for donor A, TGFβ-1 (434,461.36 pg/mL) for donor B, IL-6 (67.6 pg/mL), IL-8 (9,231.92 pg/mL), TNFα (10.97 pg/mL) and CCL2 (36,536.95 pg/mL) for donor D, whereas donor C showed lower levels of TGFβ-1 (56,366.42 pg/mL), and thus were selected for further experiments.

### HSP stimulation of the human colonic tissue does not affect the structure of the mucosa

**Figure 3:**
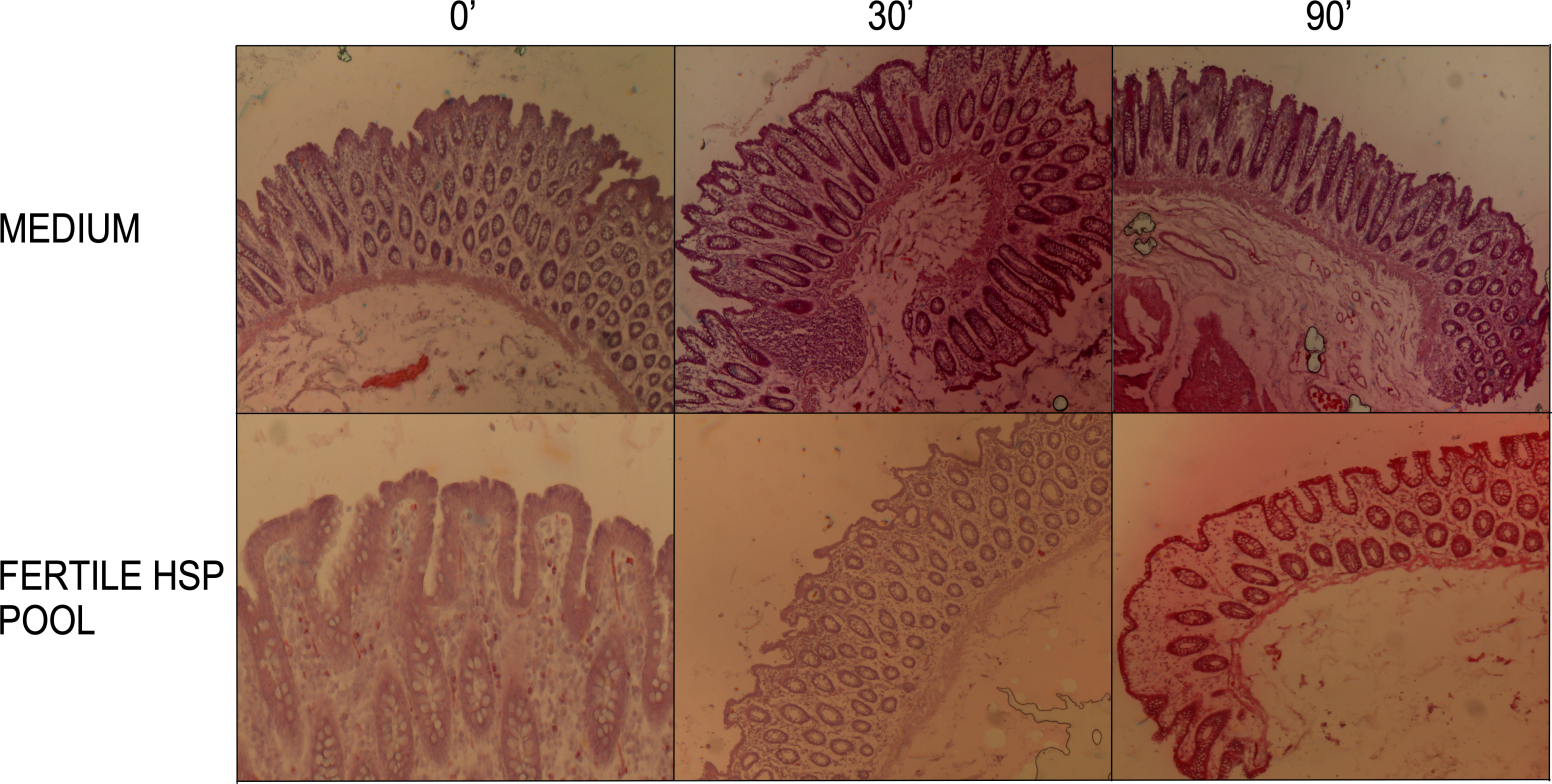
Histological analysis of colon sections. Hematoxylin-eosin staining was performed on tissues at the beginning of the experimental procedure (T0), and after 30 and 90 minutes of treatment with either medium or the HSP pool from fertile donors. Shown are 4x objective magnification. Representative images from Hematoxylin-eosin stained colon sections (10 µm thickness) showed no morphological differences in medium compared to HSP treated tissues. Preserved epithelial luminal contour, lamina propria, muscolaris mucosae and submucosa were observed in all conditions.

To verify that the colonic tissue was not altered during the experimental procedures, Hematoxylin-Eosin staining was performed on tissues at the beginning of the experimental procedure (T0), and after 30 and 90 minutes of treatment with either control medium or the HSP pool from fertile donors (**Figure 3**).

A brightfield microscope was used to analyze the overall morphology of the tissue. The *lamina propria* was preserved as well as the continuous mono-stratified columnar epithelium with a recognizable connective submucosa. In addition, the underlying muscularis mucosae was also conserved in all the analyzed biopsies of three different tissues. These observations lead us to conclude that the physiological steady conditions and the global integrity of the excised intestinal mucosa were fully preserved up to 90 minutes of stimulation.

### HSP does not perturb the human colonic barrier integrity

**Figure 4:**
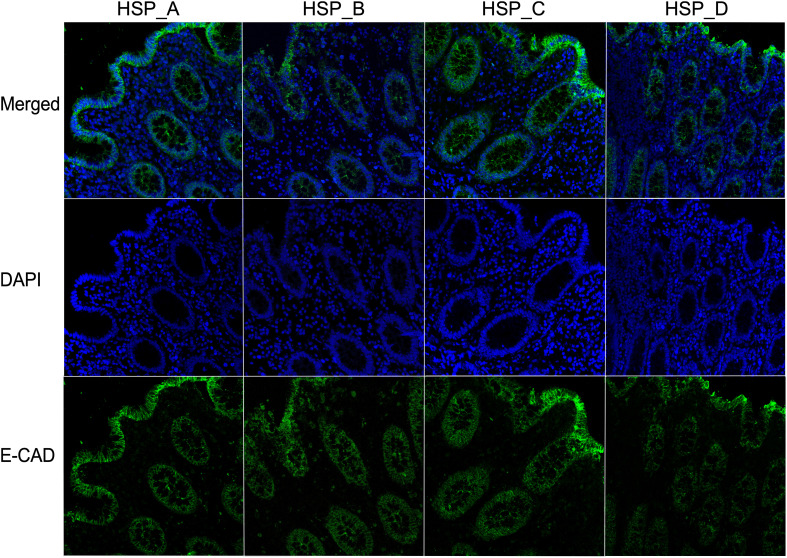
HSP from fertile donors does not affect the integrity of the colonic epithelial barrier. Representative images of the expression of E-CAD of three different colonic mucosa stimulated with HSP from four fertile donors (subject **A–D**) for 90 minutes. The E-CAD mAb was followed by Alexa Fluor 488-conjugated goat anti-mouse IgG1 antibody (green). DAPI stained the nuclei (blue). 40X magnification.

HSP of donors A, B, C and D, which displayed dissimilar concentrations of analytes within the group of fertile donors, were selected to be independently used to stimulate *ex vivo* the colonic tissue explant and to verify if they differently affected the tissue integrity. The analysis revealed that the distribution and expression pattern of E-CAD was conserved and homogeneous among all tissue samples after 90 minutes of incubation with HSP (**Figure 4**).

**Figure 5:**
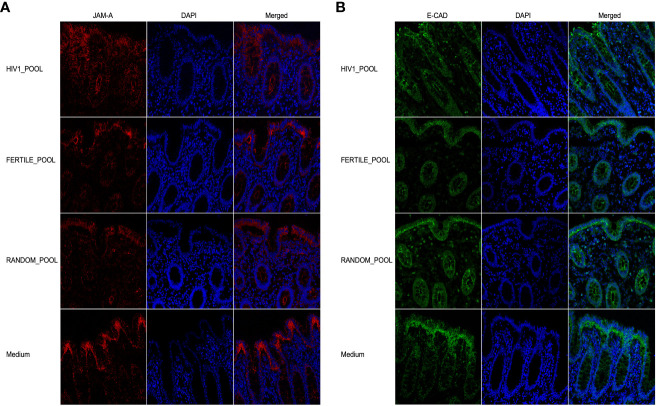
HSP does not affect colonic barrier integrity in an *ex vivo* culture model. Expression of JAM-A (red, **(A)**) and E-CAD (green, **(B)**) on tissues incubated with three HSP pools for 90 minutes, from 14 HIV infected patients (HIV1-pool), 12 HIV negative fertile subjects (Fertile-pool) or 21 randomly selected donors (Random-pool). Tight junctions were stained with the JAM-A FITC conjugated mAb, and adherent junctions with the E-CAD mAb followed by Alexa Fluor 546-conjugated goat anti-mouse IgG1 antibody. DAPI stained the nuclei (blue). 40X objective magnification are shown.

We therefore, evaluated if seminal plasma pooled from HIV-1 positive donors (HIV-load of 22,000 copies/mL), fertile HIV-negative donors, or random donors could affect the distribution of JAM-A and E-CAD proteins after 90 minutes of incubation. Our immunofluorescence and confocal microscopy analysis showed that both proteins were equally distributed and expressed in the epithelial layer of tissues stimulated with the three different pools of HSP (**Figures 5A, B**).

Our results suggest that cytokines or chemokines contained in the HSP, whether obtained from HIV-positive or negative subjects or from fertile donors, did not modulate two main proteins involved in the tight/adherent junction complex system maintaining the epithelial barrier of the colon in our *ex vivo* tissue culture model.

### HSP induces migration of CD11c+ but not CD64+ MNPs towards the epithelial layer of the human colonic mucosa

**Figure 6:**
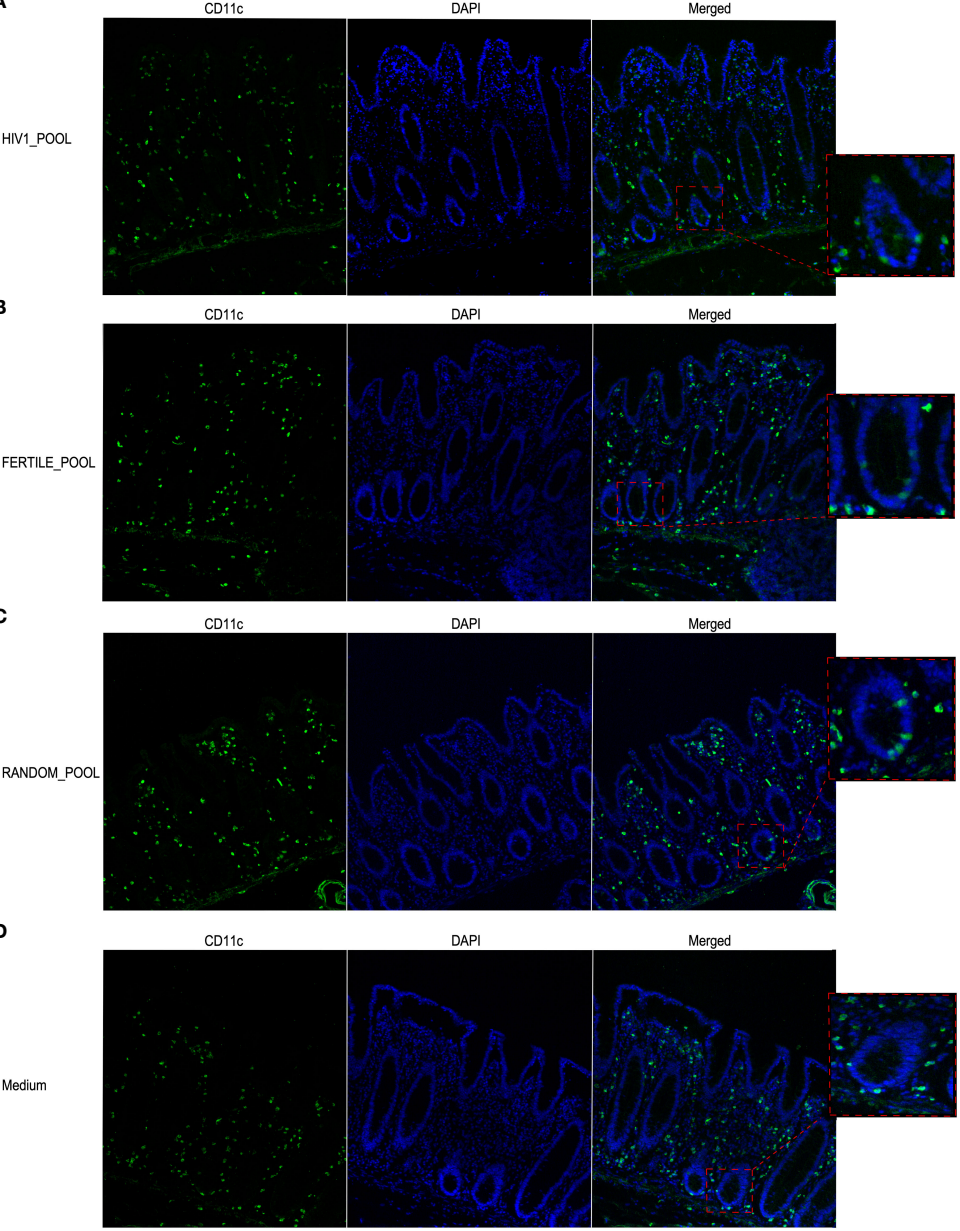
HSP recruits CD11c+ MNP cells within the intestinal epithelial layer. Representative images of CD11c+ cells localization in colonic mucosa stimulated with HSP pooled from HIV positive panel **(A)**, fertile panel **(B)**, randomly selected donors panel **(C)** or medium control panel **(D)**. Magnification of selected regions showing intraepithelial cells in A-C and subepithelial cells in D is shown in the boxed areas. Cells were stained with a rabbit anti human CD11c mAb followed by Alexa Fluor 488-conjugated goat anti-rabbit IgG antibody (green). DAPI stained the nuclei (blue).

We assessed by immunofluorescence and confocal microscopy analysis the presence and the distribution of CD11c+ MNPs (**Figure 6**) that were described to migrate into the intestinal epithelial layer in response to *ex vivo* and *in vivo* HIV stimulation in a human and non-human primate model, respectively (14, 19). After 90 minutes of tissue stimulation with HSP pooled from HIV positive (**Figure 6A**), fertile (**Figure 6B**) or random donors (**Figure 6C**), CD11c+ MNPs infiltrated within the intestinal epithelial layer of the mucosa, mostly associated with the epithelium of the crypts. A similar pattern of cells redistribution was not observed in control medium treated tissues, where CD11c+ cells were homogeneously distributed throughout the whole *lamina propria* and in proximity of the subepithelial area (**Figure 6D**).

**Figure 7:**
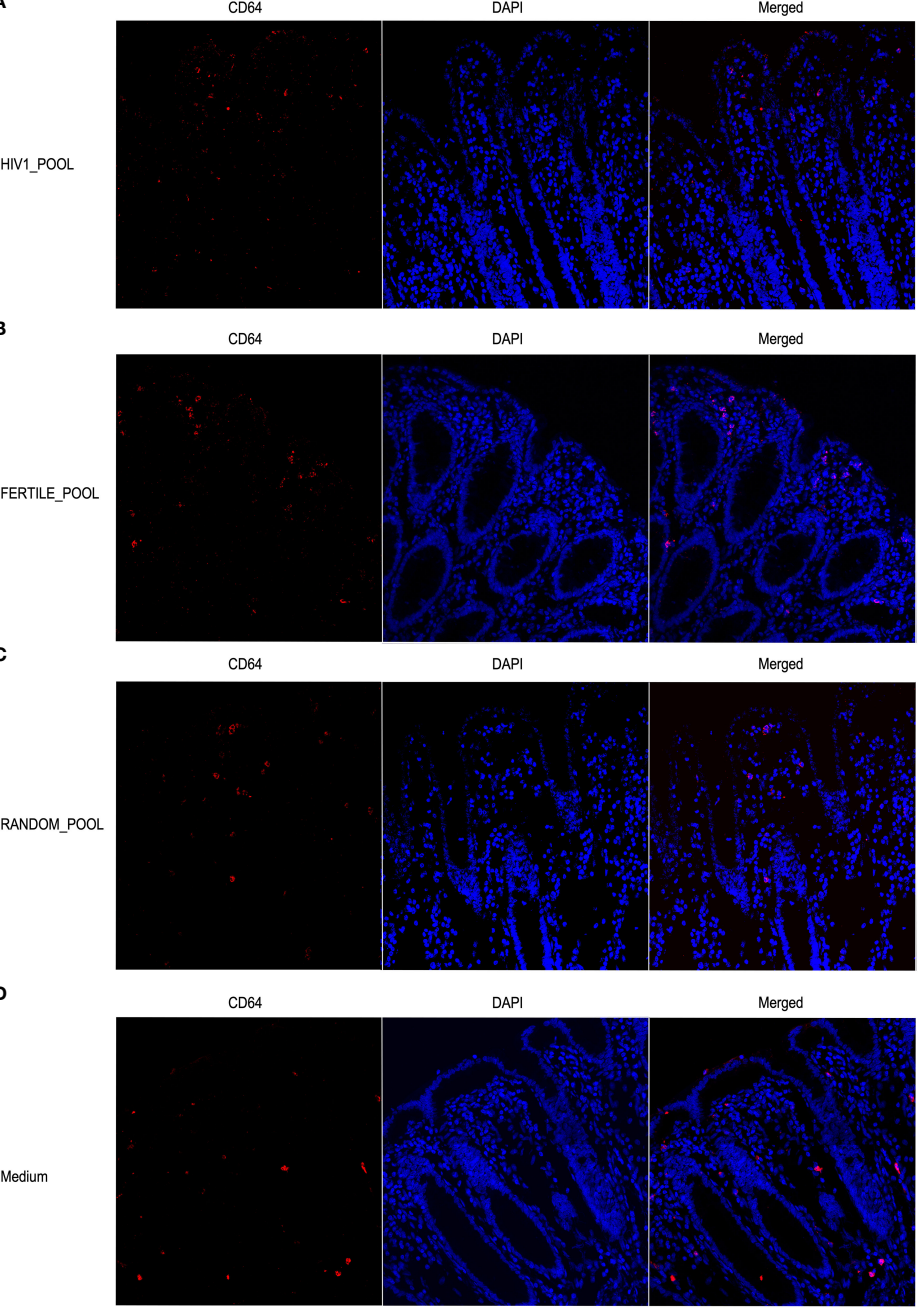
Stimulation with HSP does not induce redistribution of CD64+ MNP cells. Representative images of CD64+ positive cells show no differences in the distribution within the colonic mucosa stimulated with HSP pooled from HIV infected panel **(A)**, fertile panel **(B)**, randomly selected donors panel **(C)** or medium control panel **(D)**. Cells were stained with the mouse anti human CD64 mAb followed by Alexa Fluor 546-conjugated goat anti-mouse IgG1 antibody (red). DAPI fluorescence probe was used to detect the nucleus (blue).

We also investigated the distribution of CD64 expressing MNPs, which are putative macrophages, in the same experimental conditions. The confocal microscopy analysis showed a less abundant population of CD64+ cells than of CD11c+, that was consistently, homogeneously and uniformly distributed throughout the whole thickness of the colonic mucosa (**Figure 7**). Overall, we could not picture a clear pattern of CD64+ MNPs redistribution when the tissues were stimulated with either HSP from HIV positive (**Figure 7A**), fertile (**Figure 7B**), random donors (**Figure 7C**) or control medium (**Figure 7D**).

## Discussion

Currently, to explain how HIV crosses the intact colonic epithelium, mainly three mechanisms of translocation were proposed: paracellular diffusion, transcellular transport and immune cells mediated translocation (14, 27–29). Likely, a combined balance between these routes is used by the virus to bypass the tight epithelial layer. In our previous work we demonstrated that free HIV can attract dendritic cells to the apical side of the human colonic mucosa in an *ex vivo* tissue culture model of human colon (14, 19). Here, in this study, we showed that not only free virions but also HSP is a potent inducer of CD11c+ MNPs motility towards the luminal side of the colonic epithelium as fast as within 90 minutes of incubation. Interestingly, in our *ex vivo* tissue experiments, both HIV infected and uninfected HSP were capable to attracting CD11c+ MNPs cells in a similar fashion. The HIV load of the HSP pool of infected donors was relatively low, 20,000 copies/mL, also compared to the virus input used for the free virus experiments, thus, we cannot conclude that the virus itself of the HSP did play a prominent role in this process of attracting MNPs. However, it was already described that the viral load is usually lower in HSP than in blood, and generally detected in individuals with plasma viral loads > 10.000 cp/ml (30). Thus, regardless of the level of HSP’s viral load, we may speculate that this mechanism lays the foundation for the *in vivo* HIV capture mediated by antigen presenting cells that in turn can transfer viral particles to susceptible CD4^+^ T cells. Our findings can help to explain how HIV can be efficiently transmitted in such way even if the viral concentration is low.

HSP is a highly immunomodulatory fluid containing several bioactive molecules with the potential to influence inflammation, immune activation (31) and is thought to disturb the physical barrier of the human colonic mucosa (32, 33). We tested anti- and pro-inflammatory cytokines, chemokines and GM-CSF concentration levels of the HSP pools from HIV positive and negative donors with known fertility status to understand their role in affecting migration and permeability. As previously reported by other groups (34, 35), we observed a change in the concentration of analytes in HSP pooled from HIV infected individuals when compared to that pooled from uninfected fertile donors. In addition, we observed relevant differences in the concentrations in the HSP pooled from fertile donors compared to those with limited fertility. Unfortunately, we do not know the fertility status of the HIV-infected donors, and thus cannot conclude on the impact of the viral infection or else on the HSP’s composition. The limitation of the statistical approach used here is that the analytes were analyzed in a pool of HSP. Indeed, this approach could potentially affect the results if a single outlier sample skewed the data. However to minimize this possibility we used a large sample size of HSP from each group of donors, including 14 from HIV-positive patients, 12 from fertile HIV negative donors, and 21 from random subjects.

Several analytes included in our analysis were described to be involved in recruiting antigen presenting cells. We showed previously that, in an *in vitro* epithelial cell-DC coculture model, the chemokine CCL5 induced DCs migration through engagement of its receptors (14). The same chemokine may also act on macrophages, which express CCR5 as well (36). Additionally, other chemokines have the potential to activate and attract innate immune cells. CCL2 attracts both macrophages and dendritic cells (37), as also IP-10 does by recruiting specifically CXCR3-positive cells (38). TGF-β1 plays a role in the recruitment of both specific inflammatory dendritic cells and anti-inflammatory macrophages to the mucosal area and GM-CSF was associated to CD11c + cell and DC recruitment (39). In our data set the HSP of HIV infected donors compared to the other groups of donors showed an increased concentration of CCL5, while a decreased one of CCL2 and CCL4, other ligands of the CCR5. Regardless of the different composition of the HSP pools, in our experimental setting we did not observe differences in the capacity to recruiting MNPs or altering the permeability. We are aware that HSP pools do not allow to clearly identify the relevant analytes, however, it does not affect the overall massage that HSP induces migration of CD11+ but not CD64+ MNPs.

In addition, we showed that all HSP pools or from single donors had no disruptive impact on junctional complexes that regulate the gut paracellular permeability. Indeed, HSP did not alter the distribution of JAM-A and E-CAD in our *ex vivo* culture system when incubated as long as 90 minutes. Specifically, we highlighted an increased concentration in the HSP of HIV infected donors of pro-inflammatory cytokines like IL-1β and IFNγ, which were already described to be involved in immune cell recruitment and activation, and perturbation of the paracellular permeability (40). IL-1β and IL-15 were shown to increase intestinal permeability by promoting inflammation and decreasing the expression level of occludins (41). Contradictory results were reported regarding the role of IL-1β in modulating the expression of claudins, involved in the homeostasis of the tight junctions. In *in vitro* experiment, IFNγ enhanced the redistribution of tight junction proteins, including JAM-A, while IL-15 is involved in tight junction’s assembly (42). The difference with our results could be explained by the different experimental setting that we used: we performed experiments in explants of human intestinal mucosa that retain their *in situ* conditioning while the above cited studies used cell lines only representative of the human colonic epithelial barrier.

Overall, our findings showed that HSP plays a pivotal role in recruiting CD11c+ MNPs at the apical side of the colonic epithelium and, when infected, serves as a vector for the virus to get in close contact with the colonic mucosa and relevant cells. Cavarelli and Baharlou, with two different techniques, highlighted how free HIV initially infects dendritic cells (14, 43), and postulated that these cells are responsible for the subsequent viral spread to surrounding CD4+ T cells in the intestinal mucosa (44, 45). In sexual transmission virions, however, are carried by semen and seminal fluid, and are rarely found in free form adjacent to colonic epithelial tissue. Considering that, if parental transmission is excluded, most HIV infections are acquired through the mucosal surfaces of the genital and gastrointestinal tracts (46), strategies to prevent infection through this route must be based on a thorough understanding of the mechanism underlying the early steps of HIV mucosal infection. Our work, adds an important piece to the understanding of the initial steps of infection *via* the anogenital route, showing that the mechanism of transmission is probably made even more effective because the initial target cells of HIV are also attracted to the intestinal epithelium by seminal plasma alone.

## Data availability statement

The raw data supporting the conclusions of this article will be made available by the authors, without undue reservation.

## Ethics statement

The studies involving human participants were reviewed and approved by Ethics Committee IRCCS Ospedale San Raffaele, Milan, Italy. The patients/participants provided their written informed consent to participate in this study.

## Author contributions

VI has performed most of the *ex vivo* culture experiments. MB analyzed the data. MC set up the *ex vivo* colon tissue culture system and provided scientific expertise. CF supervised the microscopy analysis. PV, UE, SN, MA, AS provided biological material of the donors. CM and GS initiated the project and GS supervised the work. VI and MB wrote the manuscript. All authors contributed to the article and approved the submitted version.
